# Supplementary material for: Riboceine Rescues Auranofin-Induced Craniofacial Defects in Zebrafish
Source: Antioxidants (Basel). 2021 Dec 8;10(12):1964. doi: 10.3390/antiox10121964 (PMC8750187; doi:10.3390/antiox10121964)
Supplement: Supplementary file 1 [file antioxidants-10-01964-s001.zip › antioxidants-1477808-supplementary.pdf]

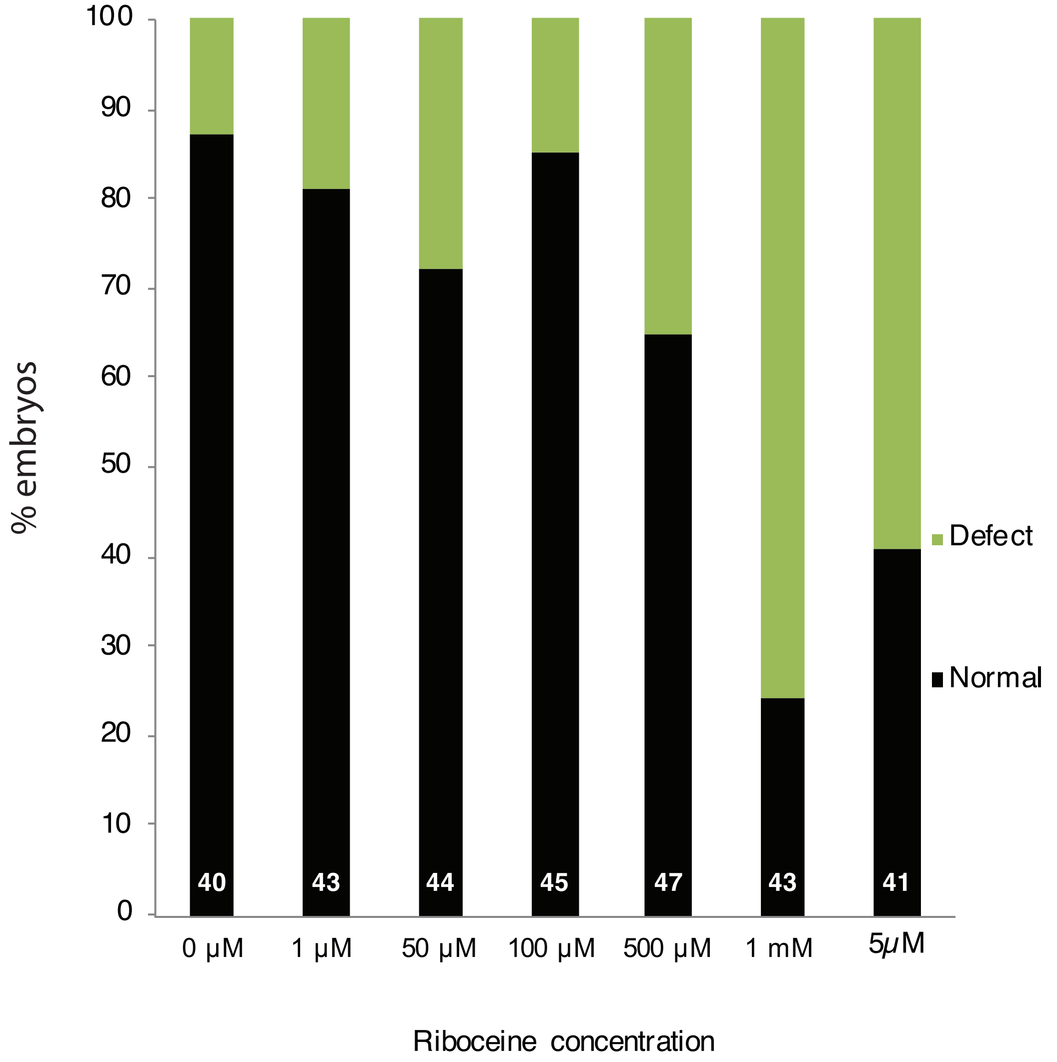

Supplementary Figure S1. Percentage of larvae with normal cranial cartilage and cranial cartilage defects in zebrafish larvae treated with increasing doses of RBC. 500 µM RBC was the highest dose before RBC became toxic. Data labels show the number of larvae observed in each treatment group. Riboceine, RBC.

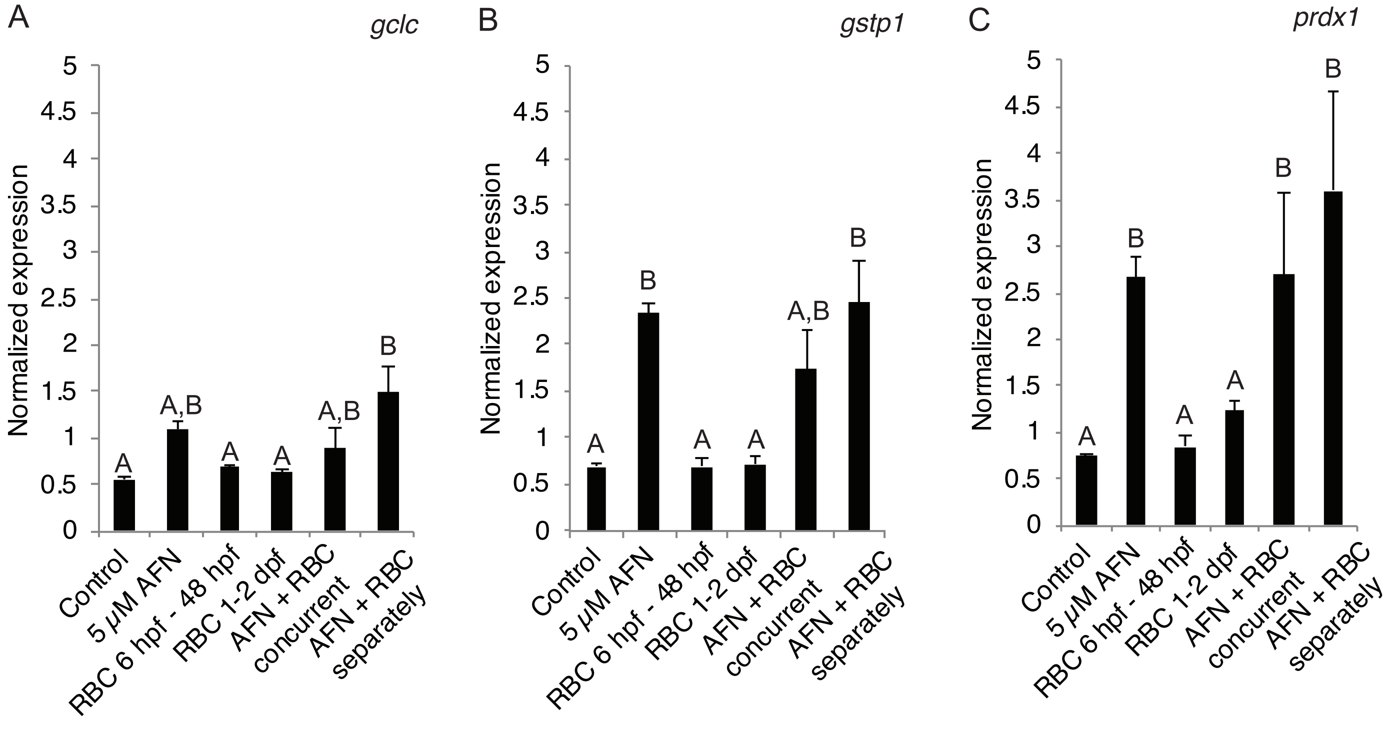

Supplementary Figure S2. Normalized expression levels of *gclc, gstp1* and *prdx1*. Normalized expression in 48 hpf embryos for gclc (A), gstp1 (B) and prdx1 (C) in treatments groups; untreated controls, 5 μM AFN, RBC (500 μM) exposure 6 hpf - 48 hpf, RBC (500 μM) expo-sure 1 - 2 dpf, 5 μM AFN and concurrent RBC (500 μM) exposure (6 hpf - 48 hpf), 5 μM AFN and separate RBC (500 μM) exposure 1-2 dpf. Error bars are standard error of the mean from three biological replicates (total n = 50 embryos). Bars not sharing a letter are statistical-ly significantly different ($p < 0.05$, one-way ANOVA, Tukey HSD post-hoc analysis). Auranof-in, AFN. Riboceine, RBC. hours post fertilization, hpf. days post fertilization, dpf.
